# Retraction Note: Colloidal silver diphosphide (AgP_2_) nanocrystals as low overpotential catalysts for CO_2_ reduction to tunable syngas

**DOI:** 10.1038/s41467-022-33833-5

**Published:** 2022-10-27

**Authors:** Hui Li, Peng Wen, Dominique S. Itanze, Zachary D. Hood, Xiao Ma, Michael Kim, Shiba Adhikari, Chang Lu, Chaochao Dun, Miaofang Chi, Yejun Qiu, Scott M. Geyer

**Affiliations:** 1 Department of Chemistry, Wake Forest University, Winston-Salem, NC 27106 USA; 2 Shenzhen Engineering Lab of Flexible Transparent Conductive Films, School of Materials Science and Engineering, Harbin Institute of Technology, Shenzhen, 518055 China; 3 Center for Nanophase Materials Sciences (CNMS), Oak Ridge National Laboratory (ORNL), Oak Ridge, TN 37831 USA; 4 Department of Materials Science and Engineering, Massachusetts Institute of Technology, Cambridge, MA 02139 USA; 5 Material Science and Technology Division (MSTD), Oak Ridge National Laboratory (ORNL), Oak Ridge, TN 37831 USA; 6 Department of Aerospace and Mechanical Engineering, University of Notre Dame, Notre Dame, IN 46556 USA

**Keywords:** Electrocatalysis, Heterogeneous catalysis, Carbon capture and storage, Nanoparticles

Retraction of: *Nature Communications* 10.1038/s41467-019-13388-8, published online 16 December 2019

The authors are retracting the Article due to several issues with figure assembly and data integrity. Nanoparticles appear to be duplicated within the same image in Figure 1a. In addition, there are unexplained anomalies in Supplementary Figures 1, 2, 24, and 36. These issues undermine confidence in the integrity of the study and its conclusions. All authors agree with this retraction.

